# Supplementary figures and images for: The Combination of Molnupiravir with Nirmatrelvir or GC376 Has a Synergic Role in the Inhibition of SARS-CoV-2 Replication In Vitro
Source: Microorganisms. 2022 Jul 21;10(7):1475. doi: 10.3390/microorganisms10071475 (PMC9323947; doi:10.3390/microorganisms10071475)

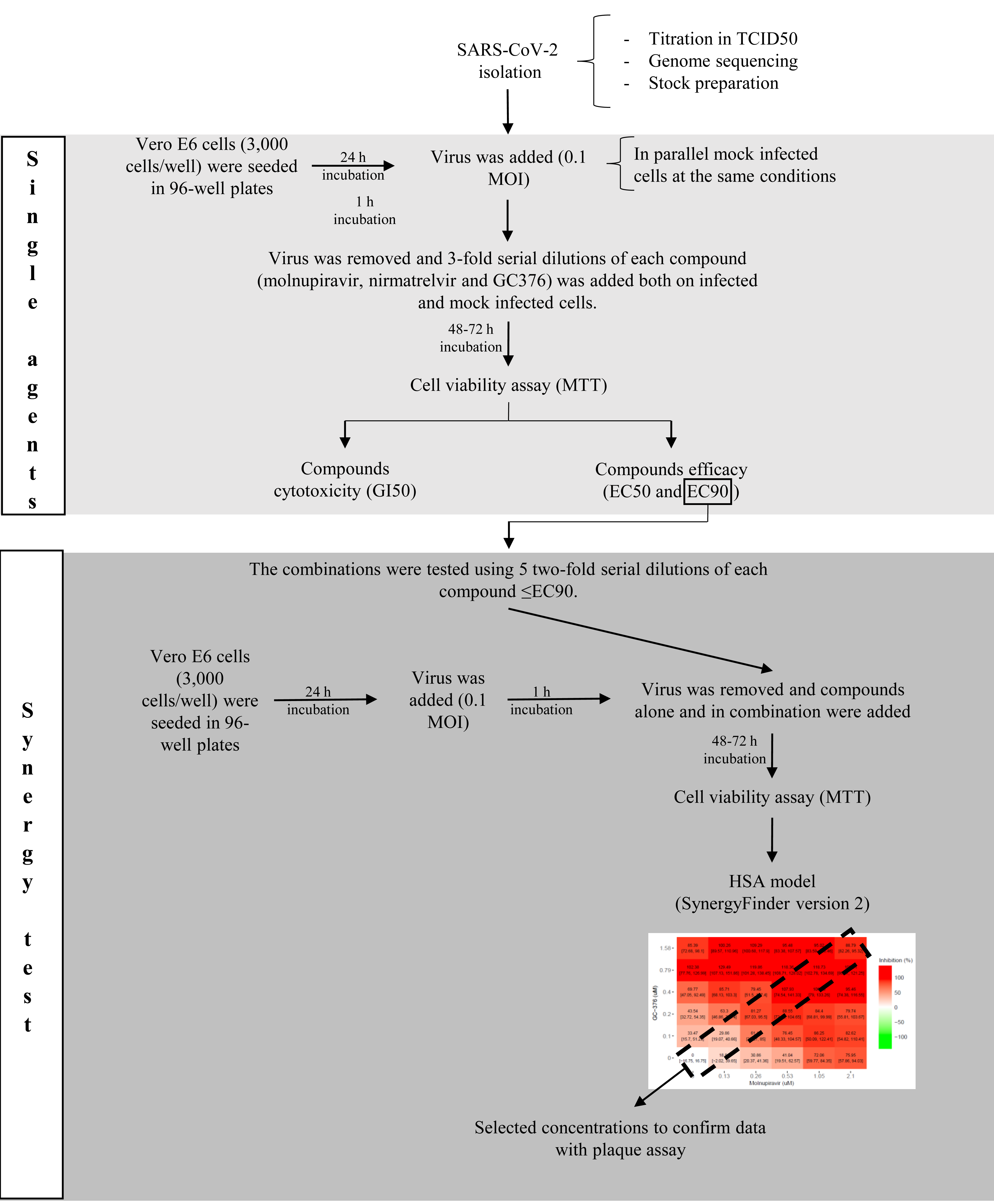

Supplement: Supplementary file 1 [file microorganisms-10-01475-s001.zip › microorganisms-1797276-supplementary Figure S1.tif]
